# Chestnut Cultivar Identification through the Data Fusion of Sensory Quality and FT-NIR Spectral Data

**DOI:** 10.3390/foods10112575

**Published:** 2021-10-26

**Authors:** Piermaria Corona, Maria Teresa Frangipane, Roberto Moscetti, Gabriella Lo Feudo, Tatiana Castellotti, Riccardo Massantini

**Affiliations:** 1 Department for Innovation in Biological, Agro-Food and Forest Systems (DIBAF), University of Tuscia, Via San Camillo de Lellis, 01100 Viterbo, Italy; piermaria.corona@unitus.it (P.C.); rmoscetti@unitus.it (R.M.); massanti@unitus.it (R.M.); 2 CREA Research Centre for Forestry and Wood, 52100 Arezzo, Italy; 3 CREA Research Centre for Olive, Fruit and Citrus Crops, 87036 Rende, Italy; gabriella.lofeudo@crea.gov.it; 4 CREA Research Centre for Agricultural Policies and Bioeconomy, 87036 Rende, Italy; tatiana.castellotti@crea.gov.it

**Keywords:** food quality assessment, non-destructive analysis, near-infrared spectrum, visible spectrum, sensory panel, Italy

## Abstract

The world production of chestnuts has significantly grown in recent decades. Consumer attitudes, increasingly turned towards healthy foods, show a greater interest in chestnuts due to their health benefits. Consequently, it is important to develop reliable methods for the selection of high-quality products, both from a qualitative and sensory point of view. In this study, Castanea spp. fruits from Italy, namely Sweet chestnut cultivar and the Marrone cultivar, were evaluated by an official panel, and the responses for sensory attributes were used to verify the correlation to the near-infrared spectra. Data fusion strategies have been applied to take advantage of the synergistic effect of the information obtained from NIR and sensory analysis. Large nuts, easy pellicle removal, chestnut aroma, and aromatic intensity render Marrone cv fruits suitable for both the fresh market and candying, i.e., marron glacé. Whereas, sweet chestnut samples, due to their characteristics, have the potential to be used for secondary food products, such as jam, mash chestnut, and flour. The research lays the foundations for a superior data fusion approach for chestnut identification in terms of classification sensitivity and specificity, in which sensory and spectral approaches compensate each other’s drawbacks, synergistically contributing to an excellent result.

## 1. Introduction

The genus *Castanea* belongs to the Fagaceae family and is found in southern Europe, eastern North America, northern Africa, Asia Minor, and eastern Asia. Castanea species show high levels of genetic diversity, and this has favored their adaptation to different environmental conditions [1]. The most important species are *Castanea sativa* (Mill.) in Europe, *Castanea dentata* (Borkh) in America, *Castanea crenata* (Sieb et Zucc.) in Japan, and *Castanea mollissima* (Blume) in China and Korea [2]. *Castanea sativa*, commonly known as European sweet chestnut, is distributed across several European countries, mainly in Italy, Spain, France, Greece, Portugal, and Turkey, with Italy having the largest area [3]. According to the Food and Agriculture Organization Statistical Database, the worldwide chestnut production is ~2.350 Tg. Chestnuts are mainly cultivated in China (1.965 Tg), Bolivia (84.00 Gg), Turkey (63.60 Gg), the Republic of Korea (53.385 Gg), and Italy (53.30 Gg) [4].

Various studies carried out about the chemical composition and nutritional characteristics of chestnut fruits confirmed that they are relatively low in calories and fat, while are rich sources of starch, trace elements, vitamins, and phytonutrients, which makes them an interesting healthy food [5,6,7,8,9,10,11,12,13,14]. Moreover, chestnut fruits do not contain gluten, so they prove to be suitable for persons suffering from celiac disease. Various compositional and health studies concluded that chestnut fruits have considerable potential as functional foods [15]. In summary, chestnuts may provide beneficial effects on health and represent a great economic resource, owing to their availability and low cost [16].

Consumer attitudes are slowly changing as demand for healthy foods is growing, resulting in an even greater interest in chestnuts due to their benefits. On the other hand, consumer awareness requires the development of reliable methods for selecting high quality products, both from qualitative and sensory points of view. The assessment of chestnut characteristics represents a crucial aspect to this end: the non-destructive measurement of quality attributes is needed so that resulting information can be exploited for improving both production and marketing systems. Indeed, increased economic interest for chestnut nuts in the food industry has fostered the demand for selected cultivars with high-quality characteristics, such as good sensorial and qualitative properties.

Recent publications prove the potential of near-infrared spectroscopy (NIR) as a rapid non-destructive method for the analysis of agro-food [17,18,19]. Additionally, NIR spectroscopy has been adopted for quality assessment of chestnuts. For instance [20], used NIR spectroscopy as a non-destructive and rapid method for determining the sugar content of chestnuts. Li Xiaoyu et al. [21] measured protein content in chestnuts by near-infrared spectroscopy, stressing the rapidity of the method when compared to the conventional methods. Hu et al. [22] tested a rapid evaluation of the quality of chestnuts by NIR diffuse reflectance spectroscopy, measuring internal mildew to classify normal and mildewed chestnuts. Other authors investigated the use of NIR spectroscopy as a rapid method to detect insect damage in chestnuts [23,24]. An analytical method for the authentication of the geographical origin of the “Vallerano” PDO chestnut by near-infrared spectroscopy was developed by Nardecchia et al. [25]. Bedini et al. [26] demonstrated the feasibility of using both conventional and imaging NIR spectroscopy for the detection of unsound chestnuts in comparison with the traditional sorting technique.

NIR spectroscopy has been successfully used to correlate different quality indices, such as acidity, ascorbic acid, soluble sugar content, dry matter, sensory characteristics for various vegetables, and fruits [27,28,29]. Several studies consider the sensory and chemical analysis of different chestnut cultivars [30,31,32,33,34,35,36,37,38] or other foods [39,40,41,42]. Nevertheless, no literature is available on the data fusion of sensory attributes or Fourier-transform Near-Infrared (FT-NIR) spectral data.

In the present study, we have applied a combination of two approaches, FT/NIR spectroscopy and sensory analysis, to describe and characterize two different chestnut cultivars in order to comprehensively assess their qualitative and sensory characteristics. We have adopted data fusion strategies for the cultivar recognition of chestnut fruits. The data fusion system is increasingly used in the fields of food quality evaluation since allows obtaining more accurate results rather than using several techniques separately [43,44,45,46,47,48].

## 2. Materials and Methods

### 2.1. Sample Preparation

Samples of *Castanea sativa* fruits (5 kg for each considered cultivar) were collected in November 2020: *Sweet chestnut* cv (C) from the collection field of the Calabria (southern Italy) and the *Marrone* cv (M) from the “Mastrogregori Company”, located in the Cimini Mountains (Central Italy). Since chestnuts can be consumed in different ways (raw, boiled, roasted, etc.) after preliminary tests, the quality of each batch was evaluated as:

− raw chestnuts (r), as in the assessment system currently adopted by traders to evaluate chestnuts; it is a quick method, used even by official inspectors for the assignment under the Protected Designation of Origin;
− boiled chestnuts (b), which represent the most widespread way of consumption; chestnuts, previously crosscut on the top, are boiled at 100 °C for 45 min [49].

The analyses were carried out on fruits from both cultivars, raw and boiled (Cr: raw Sweet chestnut; Mr: raw Marrone chestnut; Cb: boiled Sweet chestnut; Mb: boiled Marrone chestnut).

### 2.2. Fruit Weight and Morphological Attributes

Fruit weight and morphological attributes, such as length, width, thickness, geometric mean diameter, sphericity, volume, and surface area of raw chestnuts were determined [50]. In total, 15 randomly selected fruits from each cultivar (C, M) were subjected to physical assessments. Three chestnut dimensions (length, L; width, W; thickness, T) were measured by a digital vernier caliper with a resolution of 0.01 mm. Geometric mean diameter (D_g_), sphericity (Ø), arithmetic mean diameter (D_a_), surface area (S), and volume (V) were calculated by the following equations [51]:

D_g_ = (LWT)^1/3^ (1)

Ø = (LWT)^1/3^ L^−1^ (2)

D_a_ = (L + W + T) 3^−1^ (3)

S = π D_g_^2^ (4)

V = π 6^−1^ LWT (5)

### 2.3. FT/NIR Spectral Acquisition

**Figure 1:**
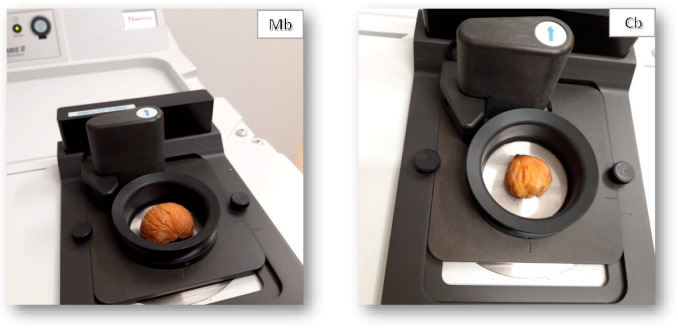
Vis-NIR spectral measurement of boiled Marrone chestnut (Mb) and boiled Sweet chestnut (Cb).

Chestnut spectra were acquired from peeled fruits of both cultivars, raw and boiled (Figure 1). The Antaris II spectrophotometer (Thermo Scientific, Madison, WI, USA) was used for the acquisition of Fourier’s transformed spectra in the 10,000–4000 cm^−1^ spectral region with a resolution of 4 cm^−1^. The instrument was allowed to warm up for at least 1 h to reach a stable state.

The spectra were acquired in diffuse reflectance mode and directly converted to absorbance by the means of the software RESULT 3 (Thermo Fisher Scientific, WI, USA). Measurements were performed at room temperature (20 ± 1 °C). Each spectrum had an average of 30 interferometer sub-scans, with the internal instrument standard as reference. A dataset of 96 samples was acquired (24 per class). Immediately after the spectra acquisition, fruits were subjected to sensory evaluation.

### 2.4. Sensory Analysis

Sensory evaluation, a technique largely applied to a wide range of food [52], was adopted to identify and quantify the raw then boiled chestnut organoleptic characteristics. Sensory analysis was conducted in a laboratory equipped for sensory analysis according to the ISO 8589 (2007) [53] standards, by eight official panelists, led by a panel leader, according to the UNI EN ISO13299 (2016) [54]. The chestnuts were washed three times in tap water. Half of them were left raw, while the other half were boiled in water at 100 °C for 45 min. Boiled chestnuts were cooled to room temperature (18 ± 2 °C) before sensory evaluation. In total, 96 samples (24 per cultivar, with two replicates) were used for the sensory analysis, randomly collected from each of the two cultivars, both raw rather than boiled. Three chestnuts of *Marrone* cv (M) and three of *Sweet chestnut* cv (C), both raw rather than boiled, were tested by each panelist during every session. Chestnuts were then placed in Styrofoam bowls and covered with a lid for 10 min before serving. Panelists were instructed to use all three samples during evaluations. Distilled, deionized water and unsalted crackers were served as palate cleansers. A minimum break of 5 min was taken between each sample. Three training sessions were carried out with the judges to ensure a common lexicon. After each panel training session, a focus group was done to decide the appropriate descriptors to use [55,56,57], and Quantitative Descriptive Analysis (QDA) was conducted as an analytical-descriptive method [49]. For raw chestnuts, ease of peeling, seed color, degree of pellicle penetration into the kernel, crunchiness, sweetness, bitterness, astringent, chestnut aroma, and aromatic persistence were the selected descriptors. For boiled chestnuts, the selected descriptors were ease of peeling, seed color, flouriness, sweetness, bitterness, saltiness, chestnut aroma, and aromatic intensity. Descriptive terms, definitions, and associated reference standards used in the sensory analysis are reported in Table 1. Each descriptor, based on bibliographical references [31,32,49], was evaluated on a continuous astructured scale with intervals from 0 (absence of the character) to 10 (maximum intensity). The same scale was used to evaluate the descriptor of personal judgement of each panelist, based on a subjective approval rating.

### 2.5. Chemometrics

#### 2.5.1. Spectral Pretreatments

Spectral data are generally subjected to mathematical transformation to remove and/or mitigate problems related to highly correlated features, noise, unwanted spectral variations, and baseline shifts, which can be detrimental to quantitative/qualitative analysis and lead to inaccurate or misleading results. In a solid matrix, such as that of raw and boiled chestnuts, the spectral unwanted variance can be related to the variability in the refractive index, morphology (e.g., surface roughness), and density of the samples. In the present study, the most common spectral pre-treatments were tested prior to chemometric analysis, i.e., Standard Normal Variate (SNV), Multiplicative Scatter Correction (MSC), and Savitzky-Golay first, second, and third derivatives (D1*f*, D2*f*, and D3*f*, respectively), with a second or third order polynomial fitted over a window of 11, 13, or 15 features. Every possible combination of spectral preprocessing was tested by further chemometrics steps, and only the best results, in terms of classification model performance, were retained.

#### 2.5.2. Data Fusion

The Data fusion model is the process of integrating data blocks from different sources into a single global model, which can lead to an improvement in a better interpretation of the results. In particular, data fusion can be performed at three levels: low, medium, and high [58]. In the low-level, a single matrix is created that includes all the raw data of the analyzed sources. In the mid-level, the data obtained are analyzed separately and relevant characteristics are extracted from each information block. In the high-level, the information is analyzed separately, a model is generated for each block of data, and then the responses are combined for a final fused response. In the present study, preprocessed spectra were fused at a low-level with sensory data, and the resulting matrix was autoscaled before model development.

#### 2.5.3. Classification Model Development

Spectral-based, sensory-based, and data-fusion-based classification models for raw and boiled fruits were individually developed using Partial Least Squared Discriminant Analysis (PLS-DA). PLS-DA is a supervised classification technique that applies the PLS algorithm to calculate the probability of a sample belonging to a certain class [59]. As PLS performs dimensional reduction, each model was cross-validated to select the optimal number of latent variables (LVs) capable to circumvent under-/over-fitting issues. For the intended purpose, the Root Mean Squared Error (RMSE) calculations were employed using a venetian blinds cross-validation with 10 data splits (1 sample per split).

The classification performance of each PLS-DA model was evaluated in terms of sensitivity, selectivity, and accuracy rates. The sensitivity rate represents the number of correctly classified samples in the considered class over the total number of samples in that class (Equation (6)). The selectivity rate corresponds to the number of correctly classified samples in the other classes over the total number of samples in that class (Equation (7)). The accuracy rate is the proportion of the true results in the batch (Equation (8)).

Sensitivity rate = True Positives (True Positives + False Negatives)^−1^ (6)

Selectivity rate = True Negatives (False Positives + True Negatives)^−1^ (7)

Accuracy rate = (True Positives + True Negatives) (Total Positives + Total Negatives)^−1^ (8)

Metrics were computed for calibration (CA) and cross-validation (CV) sets.

### 2.6. Data Handling and Statistical Analysis

One-way analysis of variance (ANOVA) was performed to evaluate statistical differences in the physical properties of the cultivars. The Tukey’s pairwise comparison method was performed, as well as the Honestly Significant Difference (HSD) (*p* ≤ 0.05). Results were reported as the mean, standard deviation of the mean, and coefficient of variation. The Kruskal-Wallis test was applied to sensorial data because it is non-normally distributed.

Data handling, as well as parametric and non-parametric statistical tests, were performed using R software 4.0.3, while spectral pretreatments, data fusion, and chemometrics were computed using Matlab 2017b (Mathworks, Natick, MA, USA) coupled with PLS_Toolbox software 7.5.3 (Eigenvector Research Inc., Wenatchee, WA, USA).

## 3. Results and Discussion

### 3.1. Fruit Weight and Morphological Attributes

Fruit weight and morphological attributes of the two chestnut cultivars were found to be statistically different (Table 2). The largest fruit on average and the highest values of fruit weight, length, width, and thickness, as well as geometric mean diameter, sphericity, arithmetic mean diameter, surface area, and volume, were observed in the M chestnuts. The high sphericity value is indicative of the tendency of the shape towards a sphere. Taken with the high value of sphericity in M samples, it may be deduced that these chestnuts undergo a combination of rolling and sliding actions on their flat surfaces. Hence, data concerning size and shape attributes are important in the design of the equipment for processing, harvesting, transportation, and storage [60]. The size and shape attributes of M chestnuts, such as large nut size, represent qualities suitable for fresh market and candying, i.e., marron glacé. On the other hand, due to their morphological characteristics, C fruits have the potential to be exploited for secondary food products, such as jam, mash chestnuts, and flour.

Chestnut fruits are commonly sorted in the industry according to their size and shape before processing. Thus, describing chestnut shape and size is fundamental for a range of different industrial applications (e.g., marron glacé).

### 3.2. Sensory Analysis

**Figure 2:**
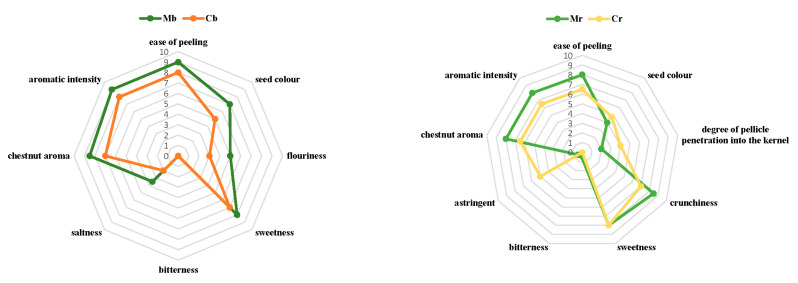
Radar chart for sensory analysis of considered chestnut varieties. Cr: raw Sweet chestnut; Mr: raw Marrone chestnut; Cb: boiled Sweet chestnut; Mb: boiled Marrone chestnut.

For raw chestnuts, Mr received a higher score by the subjective judgment of panel, with respect to Cr (8.5 and 7 of median values, respectively). Significative difference (*p* < 0.05) in subjective judgment was also found for boiled chestnuts (9 and 7 median values for Mb and Cb, respectively) (data not shown). A sensory profile of raw and boiled samples is reported in Figure 2. Both *Marrone* cv and *Sweet chestnut* cv showed significant differences (*p* < 0.05) for all the sensory descriptors (Table 3).

These results are in agreement with those by Yang et al. [38], who concluded that the sensory characteristics are significantly influenced by the cultivar of chestnut. Regarding the raw chestnuts, the ease of peeling, crunchiness, chestnut aroma, and aromatic intensity of Mr received higher scores than Cr. On the contrary, astringent and degree of pellicle penetration into the kernel of Mr was lower than that of Cr. Hwang et al. [61], who reported that chestnut pellicle peelability was negatively related to the tannin content in the edible part. In support of the above-referenced finding, our results showed high astringent values in Cr (with a score at or near 5.0) compared to those of Mr (with a score at 0.5) was the easiest to peel (pellicle removal). This aspect is particularly important since the interactions between high tannin content and a stronger astringent sensation [62] could be linked to the difficultness of pellicle removal. On the other hand, easy peeling (for fresh market and processing) and a low degree of penetration of the seed coat into the kernel (for fresh market) are appreciated qualities of chestnuts [63]. Moreover, easy pellicle removal and aromatic intensity are also all valuable traits that make this variety desirable for the industrial production of candied marrons, i.e., marrons glacés [64]. According to Poljak et al. [65], easy pellicle removal and aromatic intensity with tasty flavor render Marrone cv nuts suitable for both the fresh market and confectionery for candying and also for the production of cooked chestnuts. Although Sweet chestnut cv fruits are described as having a poorer aromatic intensity, they are suitable for fresh consumption and flour production due to achieved high values in terms of sweetness score (8 median value), according to Beccaro et al. [49]. The crunchiness attribute has a large impact on the quality of raw fruits since the release of energy during the rupture of the nut produces the characteristic sound when it is bitten and chewed, which is positively considered from a sensory perspective [66]. Concerning this attribute, Mr was crunchier than Cr, while both had the same sweetness (Figure 2).

After boiling, significant differences (*p* < 0.05) were observed between Marrone cv and Sweet chestnut cv for all the sensory descriptors. Mb presented a higher value of ease of peeling compared to Cb (9 and 8 median values, respectively), which was in agreement with other studies [30,32]. Sensory analysis on boiled samples partially confirmed the results published in previous studies [49]. Mr chestnuts showed the highest ratings for aromatic intensity, chestnut aroma, saltiness, flouriness, and seed color. The intensity of sweetness was also considerably high in Cb.

### 3.3. Overview of Spectra

**Figure 3:**
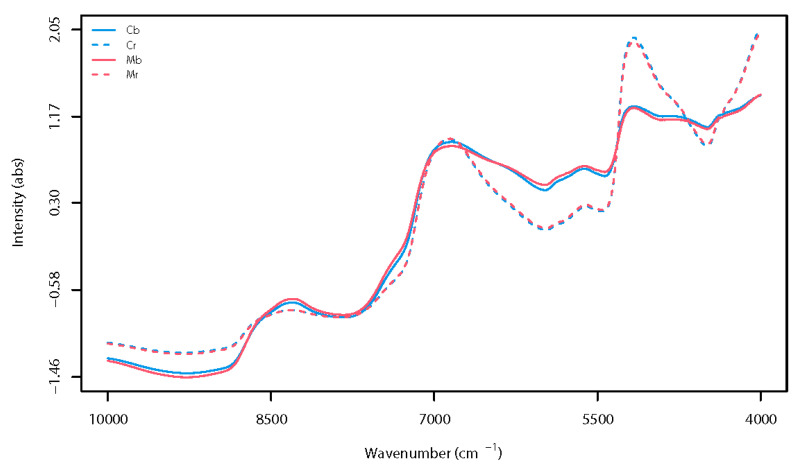
Mean absorbance spectra for raw Marrone chestnut (Mr), raw Sweet chestnut (Cr), boiled Marrone chestnut (Mb), and boiled Sweet chestnut (Cb). Spectral pre-treatment consisted of the Standard Normal Variate scatter correction, followed by the Savitzky-Golay noise filter of first polynomial order with 15 smoothing points.

Figure 3 illustrates the mean absorbance spectra of both raw and boiled kernels from Marrone and Sweet chestnut cultivars in the full range of 10,000–4000 cm^−1^. The spectra were mathematically transformed using the best pre-treatments identified during chemometrics, which specifically consisted of SNV in combination with a Savitzky-Golay smoothing filter of a first polynomial order over a window of 15 points, which means that performances of models developed using raw spectra were negatively affected by the additive and/or multiplicative effect of light scattering on the fruit surface, as well as the spectral noise. In other words, the described spectral pre-treatments helped circumvent that issues reducing the negative impact of the noninformative variance of spectra on model performances. As expected, the visual overview of the spectra showed differences in spectra profiles between raw and boiled chestnut kernels. The cooking process was, in fact, responsible for molecular changes in fruit matrix affecting spectral profile, regardless of the cultivar. On the other hand, a by-eye analysis of spectra did not really help distinguish kernels from the two considered varieties when belonging to the same type of matrix (i.e., raw or boiled chestnut). According to several research studies [23,24,67], within the 1000–2500-nm NIR spectral region (corresponding to 10,000–4000 cm^−1^), the moisture content of chestnut and foods is related to peaks of [i] the O-H stretching and bending combination at 1190 nm (~8403 cm^−1^), [ii] the O-H stretch first overtone at 1450 nm (~6896 cm^−1^), and [iii] the O-H stretching and bending combination at 1940 nm (~5155 cm^−1^). The last was the most affected by the boiling process, resulting in lower absorbance in cooked chestnuts, regardless of the cultivar. In addition, other spectral bands that evidenced changes due to cooking were identified at [iv] 1130–1160 nm (8840–8655 cm^−1^), corresponding to the first overtone of C–H stretching of the starch [68] and [v] 1700-nm and 2300-nm areas (5882 and 4348 cm^−1^), which are both related to N-H, C-N, and C=O stretching vibration of proteins, starch, and fibers [22]. The observed spectral variation in boiled chestnuts can be attributed to molecular modifications of fruit due to the hydration process and changes in structural properties of chestnut proteins and starch, which occurred during boiling [68,69]. Based on the obtained results, all classification models were individually developed on raw and boiled chestnuts, and the impact of cooking on the discriminant performances of PLS-DA models from sensory and FT-NIR data, alone or data fused, was also evaluated.

### 3.4. Classification Models

In Table 4, the performance metrics are summarized for the PLS-DA models, which gave the best discriminant performance for sensory-based, spectral-based, and data-fusion-based classifications. In general, the proposed methodology ranged from fair (>0.75) to good (>0.90), very good (>0.95), and excellent (>0.99) classification performances, with models on raw chestnuts always outperforming those on boiled product, regardless of the type of data source. In almost all cases, models possessed both selectivity and sensitivity ratios higher than 0.90, except for the spectral based model on boiled fruits, characterized by a fair accuracy ratio (i.e., 0.78–0.85).

**Figure 4:**
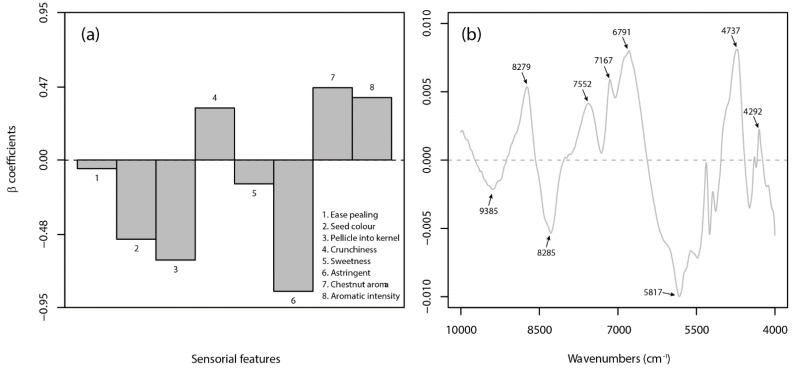
β-coefficients plots for the best sensory-based (**a**) and spectral-based (**b**) PLS-DA models on raw chestnuts.

The sensory-based PLS-DA models (Sr and Sb) led to very good and good classification results for raw and boiled products, respectively. The Sr and Sb models had accuracy rates of 0.97 and 0.95, respectively, by using three latent variables. However, the X-block cumulative variance accounted for by both models was below the ideal threshold of 90%. This implies that several sensory data were not well correlated with the categorial variable (or class label, Y), probably due to the inherent characterizes of the sensory analysis. The evaluation of the β-coefficients for the Sr model showed that 6 out of 8 attributes were the most informative (Figure 4a).

**Figure 5:**
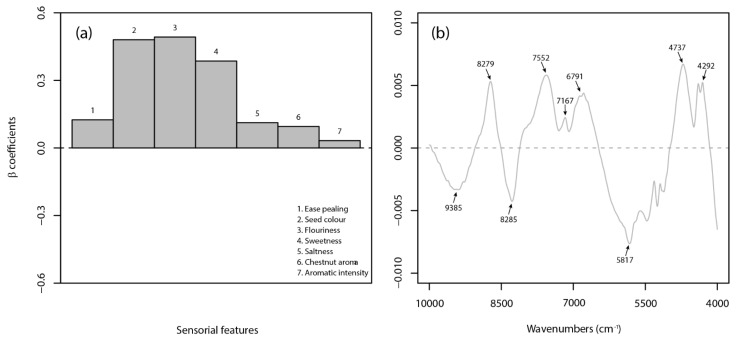
β-coefficients plots for the best sensory-based (**a**) and spectral-based (**b**) PLS-DA models on boiled chestnuts.

In detail, the Marrone cultivar was negatively related to ‘seed color’, ‘degree of pellicle into the kernel’, and ‘astringency’ and positively linked to ‘crunchiness’, ‘chestnut aroma’, and ‘aromatic intensity’ attributes. Both ‘ease pealing’ and ‘sweetness’ traits had a negligible effect on cultivar recognition. Distinctively for the boiled chestnut, the most important features for the Sb model were identified in 3 out of 7 descriptors (i.e., ‘seed color’, ‘flouriness’, and ‘sweetness’), which were positively related to the Marrone cultivar (Figure 5a). The performance of the spectral-based PLS-DA models varied significantly according to the type of matrix (i.e., raw or boiled chestnut). Very good results were obtained on the raw product (i.e., Nr model) with consistent sensitivity, selectivity, and accuracy ratios of 0.98, demonstrating the feasibility of using NIR spectroscopy for the authentication of raw peeled chestnuts, in agreement with findings obtained on in-shell fruits by Nardecchia et al. [25]. On the other hand, the classification of cultivars in boiled products did not seem an easy task for a spectral-based approach. The Nb model had an accuracy of 0.85 and 0.78 in calibration and cross-validation, respectively, which, as well as indicating a lower discrimination capability, highlights a lack of robustness. The β-coefficients (Figure 4b and Figure 5b, respectively) surprisingly showed evident similarities between models (Nr and Nb) in identifying the most contributing wavebands to the classification task. In both cases, the identified NIR bands were assigned to the combination band of the second overtone of OH stretching with the fundamental band of CH stretching (9386 cm^−1^), the second overtone of C-H stretching (8729 and 8285 cm^−1^), the CH stretching and deformation combination (7552 and 7167 cm^−1^), the second overtone of NH stretching (6791 cm^−1^), the spectral region of the CH stretching (5817 cm^−1^), the OH bending and CO stretching combination (4737 cm^−1^), and the CH stretching and CH_2_ deformation combination (4292 cm^−1^) [70]. In general, as already mentioned, the wavebands, ranging from about 10,000 to 5900 cm^−1^, are mainly related to carbohydrates and moisture content, while at 5900–4000 cm^−1^, the functional groups of proteins, starch, and fibers are more represented, as well as overlapped with the water peak located at 5200 cm^−1^.

All fusion-based PLS-DA models (Fr and Fb) led to impressive classification results, showing clear improvements, with respect to both sensory-based and spectral-based approaches. Bearing in mind the explorative approach of the present study and, thus, that further confirmatory experiments will be required (e.g., model validation with a real external dataset), the obtained performance metrics prove to be excellent, consistent, and robust. The added value of the data fusion approach is particularly evident on the authentication of the boiled product, overwhelming both Sb and Nb models. The Fb model, characterized by 7 LVs, also shows high X-block and Y-block captured variances (99.9 and 84.4%, respectively), which implies high correlation among predictors and predictand. In addition, the results demonstrate that cultivar classification on the raw peeled product does not require a data fusion approach: both Sr and Nr models are feasible; however, the spectral approach appears interesting for its fast and inexpensive nature.

## 4. Conclusions

Food quality assessment is rapidly evolving as new consumer needs arise and new techniques and tools become available. However, the exploitation of the latter, as well as their implementation within operative processes, should be evidence-based [71].

From this study, the Sweet chestnut and Marrone cvs proved to be easily distinguished by all the physical properties and sensory traits. Overall, results suggest that, due to the aforementioned differences, these two chestnut cultivars could have different practical applications. In this regard, we believe that our results will be helpful for the selection of chestnut cultivars with different sensory characteristics for various industrial applications. Large nuts, easy pellicle removal, chestnut aroma, and aromatic intensity make Marrone cv fruits suitable for both the fresh market and candying, i.e., *marron glacé*. On the other hand, due to their characteristics, Sweet chestnut fruits have the potential to be used for secondary food products, such as jam, chestnut mash, and flour. Moreover, sensory characterization as tool could also be helpful in the promotion of local chestnut cultivars and, thus, increase interest within a gastronomic tourism framework.

This study has proven the effectiveness of the data fusion approach for the cultivar authentication of boiled chestnut in terms of classification sensitivity and specificity, in which sensory and spectral approaches compensate each other’s drawbacks, synergistically contributing to an excellent result. The model obtained from the data fusion at a low-level (i.e., Fb model) has the potential to compensate for the assessors’ fatigue in the sensory method, overcoming the observed limits of FT-NIR spectroscopy in the cultivar authentication of boiled chestnuts. This evidence should be subjected to further investigations: distinctively, a larger validation sample must be used to address the additional possible variations expected from growing chestnuts in different agro-pedo-climatic conditions, in addition to other cultivars.

The potential of data fusion to separate chestnuts according to their cultivar is a relevant subject of future research, since we expect that this approach will be of great importance to consumers and commercial chestnut growers for the best choice of chestnut cultivars in a particular geographic region and, ultimately, to support the improvement of the quality of the chestnuts produced. Moreover, linking sensory attributes with NIR spectra would provide an improved or advanced strategy for manufacturers and processors for chestnut cultivars authentication and quality evaluation for an evidence-based market designation and positioning.

## Figures and Tables

**Table 1:** Descriptive terms of sensory attributes and their associated reference standards.

Descriptors	Sensory Attribute Definitions	Standards and Reference Materials
ease of peeling	ease of peeling the shell and pellicle away from the nut	different level of adherence of shell/pellicle to the nut (value 0 corresponds to hard, while value 10 corresponds to easy)
seed color	external color of the seed, after removing the pellicle	seed color with a degree of darkness (value 0 corresponds to a light color seed, while value 10 corresponds to dark)
degree of pellicle penetration into the kernel	degree of penetration of seed coat into the embryo	value 0 corresponds to a no penetration, while value 10 corresponds to strong penetration (visible > 2.0 mm)
crunchiness	amount of noise generated when the sample is chewed at a fast rate with the back teeth	value 0 corresponds to a dried apple piece, while 10 corresponds to a fresh celery piece
astringent	sensation of drying, drawing-up or puckering of any of the mouth surfaces	diluted tannic acid solution (0.06–2 mg/mL)
flouriness	amount of dry, fine, powdery particles that coat the mouth during chewing.	different level of graininess of chestnut flour (from coarse to fine)
sweetness	basic taste associated with sugar (sucrose)	diluted sucrose solution (0.5–6 g/L)
bitterness	basic taste associated with caffeine	diluted caffeine solution (0.03–0.2 g/L)
saltness	basic taste associated with salt	diluted salt solution (0.3–3 g/L)
chestnut aroma	intensity of aroma of chestnut products	taste of chestnut
aromatic intensity	characteristic flavor of chestnut at the seed break	aromatics commonly associated with chestnut

**Table 2:** Fruit weight and morphological attributes of the two chestnut cultivars.

Attribute	Cultivar	Mean	SD ^3^	CV ^4^ (%)
Weight (g)	M ^1^	16.66 a	2.78	16.70
	C ^2^	7.68 b	1.18	15.30
Length (mm)	M	40.15 a	2.61	6.49
	C	29.74 b	1.80	6.05
Width (mm)	M	30.89 a	1.41	4.55
	C	14.81 b	1.77	11.95
Thickness (mm)	M	21.82 a	2.84	13.02
	C	14.37 b	1.90	13.25
Geometric mean diameter (mm)	M	29.87 a	1.76	5.89
	C	18.42 b	1.79	9.74
Arithmetic mean diameter (mm)	M	30.95 a	1.67	5.40
	C	19.64 b	1.65	8.38
Surface area (mm^2^)	M	2809.91 a	335.54	11.94
	C	1074.99 b	202.21	18.81
Sphericity (%)	M	0.75 a	0.04	5.59
	C	0.62 b	0.04	7.10
Volume (mm^3^)	M	14,214.35 a	2583.40	18.17
	C	3384.49 b	920.04	27.18

^1^ M, Marrone cv from Cimini Mountains (Central Italy). ^2^ C, Sweet chestnut cv from Calabria (southern Italy). ^3^ SD, Standard deviation. ^4^ CV, Coefficient of variation. Different letters (a, b) indicate significant differences among samples (*p* < 0.05).

**Table 3:** Sensory attributes of boiled Marrone and Sweet chestnut cvs.

Attribute	Cultivar	Mean	Median	SD	Range	CV (%)
ease of peeling	Mb	8.94	9.00	0.13	8.5–9.0	1.43
	Cb	7.87	8.00	0.17	7.5–8.0	2.16
seed color	Mb	6.95	7.00	0.14	6.5–7.2	2.06
	Cb	4.92	5.00	0.16	4.6–5.2	3.12
flouriness	Mb	4.94	5.00	0.19	4.5–5.3	3.82
	Cb	2.94	3.00	0.17	2.5–3.3	5.55
sweetness	Mb	7.99	8.00	0.13	7.7–8.0	1.60
	Cb	7.02	7.00	0.11	6.8–7.3	1.58
saltness	Mb	3.37	3.50	0.24	3–3.8.0	6.78
	Cb	2.03	2.00	0.12	1.8–2.0	6.20
chestnut aroma	Mb	8.57	8.50	0.31	8.0–9.0	3.60
	Cb	6.95	7.00	0.16	6.5–7.2	2.23
aromatic intensity	Mb	9.03	9.00	0.25	8.7–10	2.79
	Cb	7.95	8.00	0.11	7.6–8.0	1.43
subjective judgement	Mb	8.96	9.00	0.18	8.5–9.5	2.01
	Cb	6.95	7.00	0.10	6.7–7.0	1.40

Mb, Boiled Marrone chestnut. Cb, Boiled Sweet chestnut.

**Table 4:** Summary of performance metrics for classification models that gave the best results for each computational approach (i.e., sensory-based, spectral-based, and data-fusion-based).

Type of Model	Features	Matrix Type	Model Acronym	LVs	Captured Variance (%)		Sensitivity		Specificity		Accuracy	
					X-Block	Y-Block	CA ^1^	CV ^2^	CA	CV	CA	CV
Sensory based	5	Raw	Sr	3	87.34	84.37	0.96	0.96	0.98	0.98	0.97	0.97
		Boiled	Sb	3	89.93	76.84	0.95	0.94	0.95	0.96	0.95	0.95
Spectral based	3112	Raw	Nr	3	99.39	89.90	0.99	0.98	0.97	0.98	0.98	0.98
		Boiled	Nb	3	99.53	34.91	0.90	0.82	0.80	0.74	0.85	0.78
Data fusion based	3117	Raw	Fr	3	99.28	90.03	0.99	0.98	1.00	0.99	1.00	0.99
		Boiled	Fb	7	99.93	84.40	0.98	0.99	0.99	0.99	0.99	0.99

^1^ CA, Calibration. ^2^ CV, Cross-validation.

## Data Availability

The data presented in this study are available on request from the corresponding author. The data are not publicly available due to ethical reasons.
